# Geodesic Monte Carlo on Embedded Manifolds

**DOI:** 10.1111/sjos.12036

**Published:** 2013-09-13

**Authors:** Simon Byrne, Mark Girolami

**Affiliations:** Department of Statistical Science, University College London

**Keywords:** directional statistics, geodesic, Hamiltonian Monte Carlo, Riemannian manifold, Stiefel manifold

## Abstract

Markov chain Monte Carlo methods explicitly defined on the manifold of probability distributions have recently been established. These methods are constructed from diffusions across the manifold and the solution of the equations describing geodesic flows in the Hamilton–Jacobi representation. This paper takes the differential geometric basis of Markov chain Monte Carlo further by considering methods to simulate from probability distributions that themselves are defined on a manifold, with common examples being classes of distributions describing directional statistics. Proposal mechanisms are developed based on the geodesic flows over the manifolds of support for the distributions, and illustrative examples are provided for the hypersphere and Stiefel manifold of orthonormal matrices.

## 1. Introduction

Markov chain Monte Carlo (MCMC) methods that originated in the physics literature have caused a revolution in statistical methodology over the last 20 years by providing the means, now in an almost routine manner, to perform Bayesian inference over arbitrary non-conjugate prior and posterior pairs of distributions (Gilks *et al.*, 1996).

A specific class of MCMC methods, originally known as hybrid Monte Carlo (HMC), was developed to more efficiently simulate quantum chromodynamic systems (Duane *et al.*, 1987). HMC goes beyond the random walk Metropolis or Gibbs sampling schemes and overcomes many of their shortcomings. In particular, HMC methods are capable of proposing bold long distance moves in the state space that will retain a very high acceptance probability and thus improve the rate of convergence to the invariant measure of the chain and reduce the autocorrelation of samples drawn from the stationary distribution of the chain. The HMC proposal mechanism is based on simulating Hamiltonian dynamics defined by the target distribution (see Neal (2011) for a comprehensive tutorial). For this reason, HMC is now routinely referred to as Hamiltonian Monte Carlo. Despite the relative strengths and attractive properties of HMC, it has largely been bypassed in the literature devoted to MCMC and Bayesian statistical methodology with very few serious applications of the methodology being published.

More recently, Girolami & Calderhead (2011) defined a Hamiltonian scheme that is able to incorporate geometric structure in the form a Riemannian metric. The Riemannian manifold Hamiltonian Monte Carlo (RMHMC) methodology makes proposals implicitly via Hamiltonian dynamics on the manifold defined by the Fisher–Rao metric tensor and the corresponding Levi-Civita connection. The paper has raised an awareness of the differential geometric foundations of MCMC schemes such as HMC and has already seen a number of methodological and algorithmic developments as well as some impressive and challenging applications exploiting these geometric MCMC methods (Konukoglu *et al.*, 2011; Martin *et al.*, 2012; Raue *et al.*, 2012; Vanlier *et al.*, 2012).

In contrast to Girolami & Calderhead (2011), in this particular paper, we show how Hamiltonian Monte Carlo methods may be designed for and applied to distributions defined on manifolds embedded in Euclidean space, by exploiting the existence of explicit forms for geodesics. This can provide a significant boost in speed, by avoiding the need to solve large linear systems as well as complications arising because of the lack of a single global coordinate system.

By way of specific illustration, we consider two such manifolds: the unit hypersphere, corresponding to the set of unit vectors in 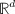, and its extension to Stiefel manifolds, the set of *p*-tuples of orthogonal unit vectors in 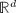. Such manifolds occur in many statistical applications: distributions on circles and spheres, such as the von Mises distribution, are common in problems dealing with directional data (Mardia and Jupp, 2000). Orthonormal bases arise in dimension reduction methods such as factor analysis (Jolliffe, 1986) and can be used to construct distributions on matrices via eigendecompositions.

The problem of sampling from such distributions has not received much attention. Most methods in wide use, such as those used in directional statistics for sampling from spheres, have been developed for the specific problem at hand, often based on rejection sampling techniques tuned to a specific family. For the various multivariate extensions of these distributions, these techniques are usually embedded in a Gibbs sampling scheme.

There are relatively few works on the general problem of sampling from manifolds. The recent paper by Diaconis *et al.* (2013) provides a readable introduction to the concepts of geometric measure theory, and practical issues when sampling from manifolds, with the motivation of computing certain sampling distributions for hypothesis testing. Brubaker *et al.* (2012), somewhat similar to our approach, develop an HMC algorithm using the iterative algorithm for approximating the Hamiltonian paths.

In the next section, we provide a brief overview of the necessary concepts from differential geometry and geometric measure theory, such as geodesics and Hausdorff measures. In section 3, we construct a Hamiltonian integrator that utilizes the explicit form of the geodesics and incorporate this into a general HMC algorithm. Section 4 gives examples of various manifolds for which the geodesic equations are known, and section 5 provides some illustrative applications.

## 2. Manifolds, geodesics and measures

### 2.1. Manifolds and embeddings

In this section, we introduce the necessary terminology from differential geometry and information geometry. A more rigorous treatment can be found in reference books such as do Carmo (1976, 1992) and Amari and Nagaoka (2000).

An *m*-dimensional manifold 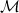 is a set that locally acts like 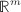: that is, for each point 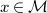, there is a bijective mapping *q*, called a *coordinate system*, from an open set around *x* to an open set in 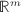. Our particular focus is on manifolds that are *embedded* in some higher-dimensional Euclidean space 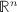, (i.e. they are submanifolds of 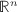). Note that 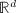 is itself a *d*-dimensional manifold, which we refer to as the *Euclidean manifold*.

*Example 2.1.* A simple example of an embedded manifold is the *hypersphere* or (*d − 1*)-*sphere*:


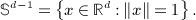


This is a (*d* − 1)-dimensional manifold, as there exists an angular coordinate system *φ* ∈ (0,2*π*) × (0,*π*)^*d* − 2^ where


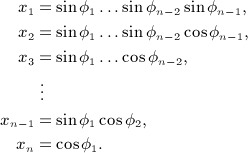


Note that this coordinate system excludes some points of 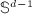: such as *δ*_*d*_ = (0, …, 0,1). As a result, it is not a *global* coordinate system (in fact, no global coordinate system for 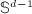 exists); nevertheless, it is possible to cover all of 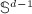 by utilizing multiple coordinate systems known as an *atlas*.

A *tangent* at a point 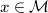 is a vector *v* that lies ‘flat’ on the manifold. More precisely, it can be defined as an equivalence class of the set of functions 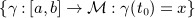 that have the same ‘time derivative’ 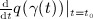 in some coordinate system *q*. For an embedded manifold, however, a tangent can be represented simply as a vector 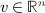 such that


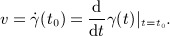


The *tangent space* is the set *T*_*x*_ of such vectors and form a subspace of 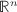: this is equal to the span of the set of partial derivatives *∂x*_*i*_ / *∂q*_*j*_ of some coordinate system *q*.

*Example 2.2.* A function on the sphere 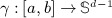 must satisfy the constraint 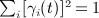. By taking the time derivative of both sides, we find that


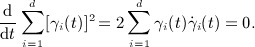


Therefore, the tangent space at 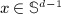 is the (*d* − 1)-dimensional subspace of vectors orthogonal to *x*:


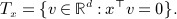


A *Riemannian manifold* incorporates a notion of distance, such that for a point 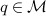, there exists a positive-definite matrix *G*, called the metric tensor, that forms an inner product between tangents *u* and *v*


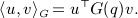


*Information geometry* is the application of differential geometry to families of probability distributions. Such a family {*p*( ⋅ ∣ *θ*) : *θ* ∈ Θ}can be viewed as a Riemannian manifold, using the *Fisher–Rao metric tensor*


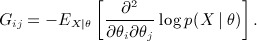


*Example 2.3.* The family of *d*-dimensional multinomial distributions


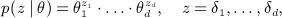


where *δ*_*i*_ is the *i*th coordinate vector, is parametrized by the unit (*d* − 1)-simplex,


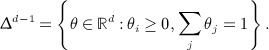


This is a (*d* − 1)-dimensional manifold embedded in 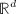 and can be parametrized in (*d* − 1) dimensions by dropping the last element of *θ*, the set of which we will denote by 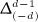.

The Fisher–Rao metric tensor in 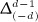 is then easily shown to be


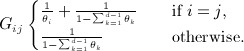


A smooth mapping from a Riemannian manifold to 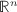 is an *isometric embedding* if the Riemannian inner product is equivalent to the usual Euclidean inner product. That is,


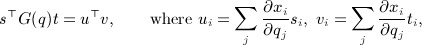


or equivalently,


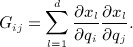
(1)

The existence of such embeddings is determined by the celebrated Nash (1956) embedding theorem; however, it does not give any guide as how to construct them. Nevertheless, there are some such embeddings we can identify.

**Fig. 1:**
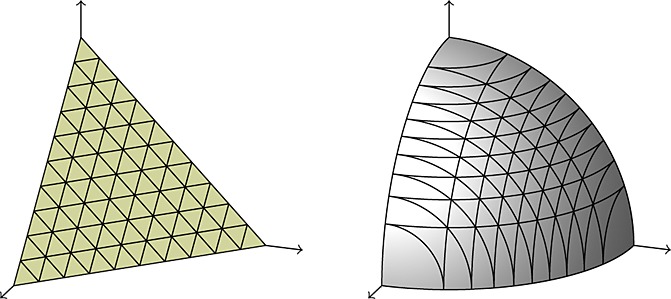
Unit 2-simplex Δ^2^ and the positive orthant of the two-sphere 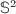. The lines on the simplex are equidistant: the transformation to the sphere stretches these apart near the boundary.

*Example 2.4.* There is a bijective mapping from the simplex Δ^*d* − 1^ to the positive orthant of the sphere 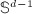 by taking the element-wise square root 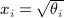 (Fig. 1). If we consider it as a mapping from 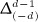, then the partial derivatives are of the form


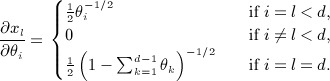


Note that by (1), this is an isometric embedding (up to proportionality) of the Fisher–Rao metric from example 2.3.

### 2.2. Geodesics

The *affine connection* of a manifold determines the relationship between tangent spaces of different points on a manifold: interestingly, this depends on the path 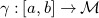 used to connect the two points, and for a vector field *v*(*t*) ∈ *T*_*γ*(*t*)_ along the path, we can measure the change by the *covariant derivative*.

Of course, the time derivative 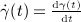 is itself such a vector field: when this follows the affine connection, the covariant derivative is 0; in which case, *γ* is known as a *geodesic*.

This property can be expressed by the geodesic equation


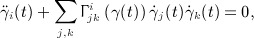
(2)

where 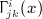 are known as the connection coefficients or Christoffel symbols. A Riemannian manifold induces a natural affine connection known as the *Levi-Civita connection*.

In the Euclidean manifold 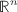, the Christoffel symbols 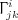 are zero, and so the geodesic (2) reduces to 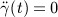. Hence, the geodesics are the set of straight lines *γ*(*t*) = *at* + *b*.

In a Riemannian manifold, the geodesics are the locally extremal paths (maxima or minima in terms of calculus of variations) of the integrated path length


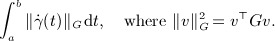


Moreover, the geodesics have *constant speed*, in that 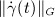 is constant over *t*. As the geodesics can be determined by the metric, they are consequently preserved under any metric-preserving transformation, such as an isometric embedding.

**Fig. 2:**
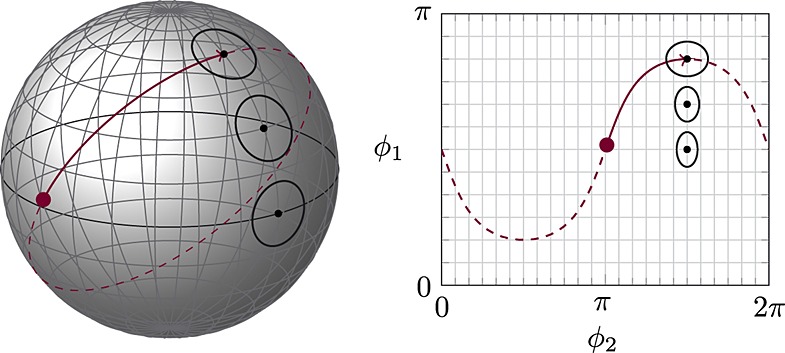
A geodesic (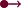) and great circle (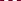) on the sphere 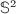 and its path in the spherical polar coordinate system *x* = (sin*φ*_1_ sin*φ*_2_,sin*φ*_1_ cos*φ*_2_,cos*φ*_1_). The ellipses correspond to equi-length tangents from each marked point.

*Example 2.5.* A standard result in differential geometry is that the geodesics of the *n*-sphere are rotations about the origin, known as *great circles* (Fig. 2):


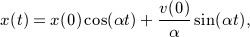


where 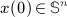 is the initial position, *v*(0) is the initial velocity in the tangent space (i.e. such that *x*(0)^⊤^ *v*(0) = 0) and *α* = ∥ *v*(0) ∥ is the constant angular velocity.

For any geodesic 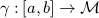, the *geodesic flow* describes the path of the geodesic and its tangent 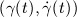. Moreover, it is unique to the initial conditions 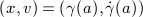, so we can describe any geodesic flow from its starting position *x* and velocity *v*: this is also known as the *exponential map*. If all such pairs (*x*,*v*) describe geodesics, then the manifold is said to be *geodesically complete*, which is true of the manifolds we consider in this paper.

### 2.3. The Hausdorff measure and distributions on manifolds

As our motivation is to sample from distributions defined on manifolds, we introduce some basic concepts of geometric measure theory that will be useful for this purpose. Geometric measure theory is a large and active topic and is covered in detail in references such as Federer (1969) and Morgan (2009). However, for a more accessible overview with a statistical flavour, we suggest the recent introduction given by Diaconis *et al.* (2013).

Our key requirement is a reference measure from which we can specify probability density functions, similar to the role played by the Lebesgue measure for distributions on Euclidean space. For this, we use the *Hausdorff measure*, one of the fundamental concepts in geometric measure theory. This can be defined rigorously in terms of a limit of coverings of the manifold (see the aforementioned references); however, for a manifold embedded in 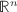, it can be heuristically interpreted as the surface area of the manifold.

The relationship between 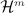, the *m*-dimensional Hausdorff measure and *λ*^*m*^, the Lebesgue measure on 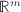, is given by the *area formula* (Federer, 1969, theorem 3.2.5). If we parametrize the manifold by a Libschitz function 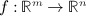, then for any 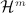-measurable function 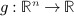,


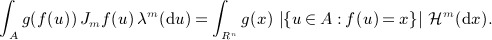


Here, *J*_*m*_*f*(*x*) is the *m*-dimensional Jacobian of *f*: this can be defined as a norm on the matrix of partial derivatives *Df*(*x*) (Federer, 1969, section 3.2.1), and if rank *Df*(*x*) = *m*, then [*J*_*m*_*f*(*x*)]^2^ is equal to the sum of squares of the determinants of all *m* × *m* submatrices of *Df*(*x*).

*Example 2.6.* The square root mapping in example 2.4 from 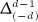 to 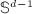 has (*d* − 1)-dimensional Jacobian


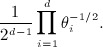


The Dirichlet distribution is a distribution on the simplex, with density


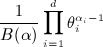


with respect to the Lebesgue measure on 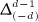. Therefore, the corresponding density with respect to the Hausdorff measure on 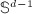 is


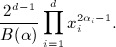


**Fig. 3:**
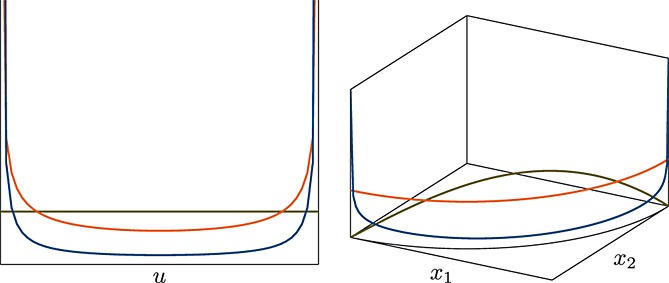
Densities of different beta (*α*,*α*) distributions for *u* ∈ (0,1) (left) and their corresponding transformations to the positive quadrant of the unit circle 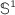, by the mapping 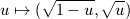 (right).*α* = 0.1 (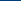), *α* = 0.5 (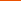) and *α* = 1.0 (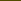).

In other words, the uniform distribution on the sphere arises when *α*_*i*_ = 1 / 2, whereas *α* = 1 gives the uniform distribution on the simplex (Fig. 3).

The area formula allows the Hausdorff measure to be easily extended to Riemannian manifolds (Federer, 1969, section 3.2.46), where


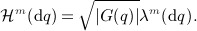


This construction would be familiar to Bayesian statisticians as the *Jeffreys prior*, in the case where *G* is the Fisher–Rao metric.

When working with probability distributions on manifolds, the Hausdorff measure forms the natural reference measure and allows for reparametrization without needing to compute any additional Jacobian term. We use 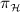 to denote the density with respect to the Hausdorff measure of the distribution of interest.

*Example 2.7.* The von Mises distribution is a common family of distributions defined on the unit circle (Mardia and Jupp, 2000, section 3.5.4). When parametrized by an angle *θ*, the density with respect to the Lebesgue measure on [0,2*π*) is


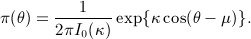


The embedding transformation *x* = (sin*θ*,cos*θ*) has unit Jacobian, so the density with respect to the one-dimensional Hausdorff measure is


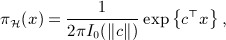


where *c* = (*κ*sin*μ*,*κ*cos*μ*) and *I*_*k*_ is the modified Bessel function of the first kind. In other words, it is a natural exponential family on the circle.

The von Mises–Fisher distribution is the natural extension to higher-order spheres (Mardia and Jupp, 2000, section 9.3.2) with density


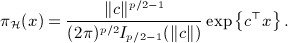


Attempting to write this as a density with respect to the Lebesgue measure on some parametrization of the surface, such as angular coordinates, would be much more involved, as the Jacobian is no longer constant.

## 3. Hamiltonian Monte Carlo on embedded manifolds

RMHMC is an MCMC scheme whereby new samples are proposed by approximately solving a system of differential equations describing the paths of Hamiltonian dynamics on the manifold (Girolami and Calderhead, 2011).

The key requirement for Hamiltonian Monte Carlo is the *symplectic integrator*. This is a discretization that approximates the Hamiltonian flows yet maintains certain desirable properties of the exact solution, namely time-reversibility and volume preservation that are necessary to maintain the detailed balance conditions. The standard approach is to use a *leapfrog scheme*, which alternately updates the position and momentum via first order Euler updates (Neal, 2011).

Given a target density *π*(*q*) (with respect to the Lebesgue measure) in some coordinate system *q*, RMHMC, Girolami & Calderhead (2011) utilize a Hamiltonian of the form


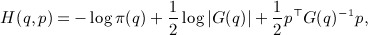


where *G* is the metric tensor. This is the negative log of the joint density (with respect to the Lebesgue measure) for (*q*,*p*), where the conditional distribution for the auxiliary momentum variable *p* is N (0,*G*(*q*)).

The first two terms can be combined into the negative log of the target density with respect to the Hausdorff measure of the manifold


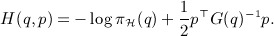
(3)

By Hamilton's equations, the dynamics are determined by the system of differential equations


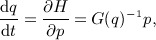
(4)


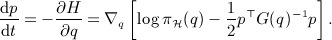
(5)

As this Hamiltonian is not separable (i.e. it cannot be written as the sum of a function of *q* and a function of *p*), we are unable to apply the standard leapfrog integrator.

### 3.1. Geodesic integrator

Girolami & Calderhead 2011) develop a generalized leapfrog scheme, which involves composing adjoint Euler approximations to ((4) and (5) in a reversible manner. Unfortunately, some of these steps do not have an explicit form and so need to be solved implicitly by fixed-point iterations. Furthermore, these updates require computation of both the inverse and derivatives of the metric tensor, which are *O*(*m*^3^) operations; this limits the feasibility of numerically naive implementations of this scheme for higher-dimensional problems. Finally, such a scheme assumes a global coordinate system, which may cause problems for manifolds for which none exist, such as the sphere, where artificial boundaries may be induced.

In this contribution, we instead construct an integrator by *splitting the Hamiltonian* (Hairer *et al.*, 2006, section II.5): that is, we treat each term in (3) as a distinct Hamiltonian and alternate simulating between the exact solutions.

Splitting methods have been used in other contexts to develop alternative integrators for Hamiltonian Monte Carlo (Neal, 2011, section 5.5.1) such as extending HMC to infinite-dimensional Hilbert spaces (Beskos *et al.*, 2011) and defining schemes that may reduce computational cost (Shahbaba *et al.*, 2011).

We take the first component of the splitting to be the ‘potential’ term


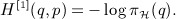


Hamilton's equations give the dynamics


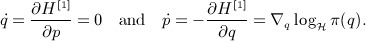


Starting at (*q*(0),*p*(0)), this has the exact solution


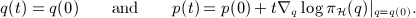
(6)

In other words, this is just a linear update to the momentum *p*.

The second component is the ‘kinetic’ term


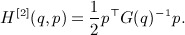
(7)

This is simply a Hamiltonian absent of any potential term, and the solution of Hamilton's equations can be easily shown to be a geodesic flow under the Levi-Civita connection of *G* (Abraham and Marsden, 1978, theorem 3.7.1) or to be more precise, a co-geodesic flow (*q*(*t*),*p*(*t*)), where 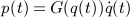.

Thus, if we are able to exactly compute the geodesic flow, then we can construct an integrator by alternately simulating from the dynamics of *H*^[1]^ and *H*^[2]^ for some time step *ε*. Each iteration of the integrator consists of the following steps, starting at position (*q*,*p*) in the phase space:

- Update according to the solution to *H*^[1]^ in (6), for a period of *ε* / 2 by setting
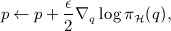
(8)
- Update according to *H*^[2]^, by following the geodesic flow starting at (*q*,*p*), for a period of *ε*.
- Update again according to *H*^[1]^ for a period of *ε* / 2 by (8).

As *H*^[1]^ and *H*^[2]^ are themselves Hamiltonian systems, their solutions are necessarily both reversible and symplectic. As the integrator is constructed by their symmetric composition, it will also be reversible and symplectic.

Therefore, the overall transition kernel for our Hamiltonian Monte Carlo scheme from an initial position *q*_0_ is as follows:

- Propose an initial momentum *p*_0_ from *N*(0,*G*(*q*_0_)).
- Map (*q*_0_,*p*_0_) ↦ (*q*_*T*_,*p*_*T*_) by running *T* iterations of the aforementioned integrator.
- Accept the *q*_*T*_ as the new value with probability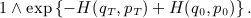  Otherwise, return the original value *q*_0_.

As with the RMHMC algorithm, the metric *G* need only be known up to proportionality: scaling is equivalent to changing the time step *ε*.

### 3.2. Embedding coordinates

The algorithm can also be written in terms of an embedding, which avoids altogether the computation of the metric tensor and the possible lack of a global coordinate system.

Given an isometric embedding 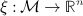, then the path *x*(*t*) = *ξ*(*q*(*t*)), such that


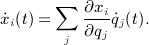


Therefore, we can transform the phase space (*q*,*p*), where 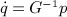, to the embedded phase space (*x*,*v*), such that


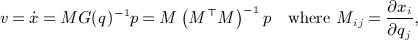


because *G* = *M*^⊤^ *M*, from (1).

By substitution, the Hamiltonian (3) can be written in terms of these coordinates as


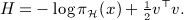
(9)

Note that the target density 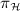 is still defined with respect to the Hausdorff measure of the manifold, and so no additional log-Jacobian term is introduced.

We can rewrite the solution to *H*^[1]^ in (6) in these coordinates. The position *x*(*t*) remains constant, and by the change of variables of the operator ∇ _*q*_ = *M*^⊤^ ∇ _*x*_, the velocity has a linear path


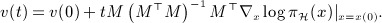


The linear operator *M*(*M*^⊤^ *M*)^− 1^*M*^⊤^ is the ‘hat matrix’ from linear regression: this is the orthogonal projection onto the span of the columns of *M*, that is, the tangent space of the embedded manifold.

Although it is possible to compute this projection using standard least squares algorithms, it can be computationally expensive and prone to numerical instability at the boundaries of the coordinate system (e.g. at the poles of a sphere). However, for all the manifolds that we consider there exists an explicit form for an orthonormal basis *N* of the *normal* to the tangent space, in which case we can simply subtract the projection onto the normal:


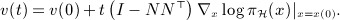


Finally, we require a method for sampling the initial velocity *v*_0_. Because *p*_0_ ∼ N (0,*G*(*q*)), it follows that


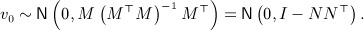


We do not need to compute a Cholesky decomposition here: because (*I* − *NN*^⊤^ ) is a projection, it is idempotent, so we can draw *z* from N (0,*I*_*n*_) and project *v*_0_ = (*I* − *NN*^⊤^ )*z* to obtain the necessary sample.

The resulting procedure is presented in Algorithm 1. In order to implement it for an embedded manifold 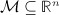, we need to be able to evaluate the following at each 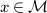:

- the log-density with respect to the Hausdorff measure 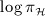, and its gradients;
- an orthogonal projection from 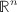 to the tangent space of 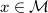;
- the geodesic flow from any 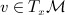.

**Table:** 

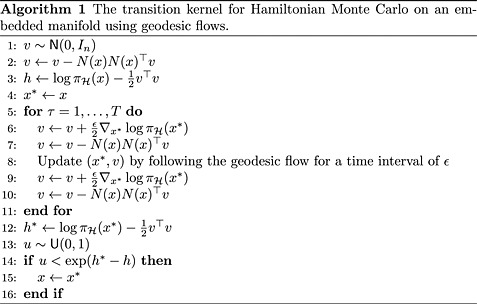

Note that by working entirely in the embedded space, we completely avoid the coordinate system *q* and the related problems where no single global coordinate system exists. The Riemannian metric *G* only appears in the Jacobian determinant term of the density: in certain examples, this can also be removed, for example by specifying the prior distribution as uniform with respect to the Hausdorff measure, as is performed in section 5.3

## 4. Embedded manifolds with explicit geodesics

In this section, we provide examples of embedded manifolds for which the explicit forms for the geodesic flow are known and derive the bases for the normal to the tangent space.

### 4.1. Affine subspaces

If the embedded manifold is flat, for example an affine subspace of 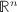, then the geodesic flows are the straight lines


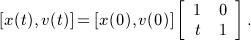


In the case of the Euclidean manifold 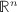, then the normal space to the tangent is null, and no projections are required. Hence, the algorithm reduces to the standard leapfrog scheme of HMC.

In standard HMC, it is common to utilize a ‘mass’ or ‘preconditioning’ positive-definite matrix *M*, in order to reduce the correlation between samples, especially where variables are highly correlated or have different scales of variation. This is directly equivalent to using the RMHMC algorithm with constant a Riemannian metric, or our geodesic procedure on the embedding of *x* = *L*^⊤^ *q*, where *L* is a matrix square root such that *LL*^⊤^ = *M* (such as the Cholesky factor).

### 4.2. Spheres

Recall from earlier examples that the unit (*d* − 1)-sphere 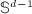 is an (*d* − 1)-dimensional manifold embedded in 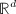, characterized by the constraint


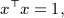


with tangent space


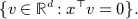


Distributions on spheres, particularly 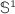 and 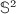, arise in many problems in directional statistics (Mardia and Jupp, 2000): examples include the von Mises–Fisher distribution (example 2.7) and the Bingham–von Mises–Fisher (BVMF) distribution (section 5.1). For many of these distributions, the normalization constants of the density functions are often computationally intensive to evaluate, which makes Monte Carlo methods particularly attractive.

As mentioned in example 2.5, the geodesics of the sphere are the great circle rotations about the origin. The geodesic flows are then


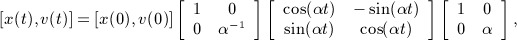
(10)

where *α* = ∥ *v*(*t*) ∥ is the (constant) angular velocity. The normal to the tangent space at *x* is *x* itself, so (*I* − *xx*^⊤^ )*u* is an orthogonal projection of an arbitrary 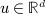 onto the tangent space.

Other than the evaluation of the log-density and its gradient, the computations only involve vector–vector operations of addition and multiplication, so the algorithm scales linearly in *d*.

### 4.3. Stiefel manifolds

A *Stiefel manifold*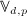 is the set of *d* × *p* matrices *X* such that


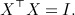


In other words, it is the set of matrices with orthonormal column vectors, or equivalently, the set of *p*-tuples of orthogonal points in 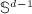, and is a 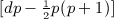-dimensional manifold, embedded in 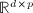. In the special case where *d* = *p*, the Stiefel manifold is the *orthogonal group*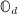: the set of *d* × *d* orthogonal matrices.

These arise in the statistical problems related to dimension reduction such as factor analysis and principal component analysis, where the aim is to find a low dimensional subspace that represents the data. They can also arise in contexts where the aim is to identify orientations, such as projections in shape analysis, or the eigendecomposition of covariance matrices.

Previously suggested methods of sampling from distributions on Stiefel manifolds, such as Hoff (2009) and Dobigeon & Tourneret (2010), have relied on column-wise Gibbs updates. Such an approach is limited to cases where the conditional distribution of the column has a conjugate form and requires the computation of an orthonormal basis for the null space of *X*, requiring *O*(*d*^3^) operations.

Again, we can find the constraints on the phase space by the time derivative of the constraint for an arbitrary curve *X*(*t*) in 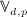



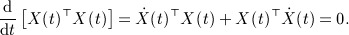


That is, the tangent space at *X* is the set


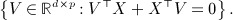


If we let 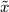 denote the matrix *X* written as a vector in 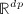 by stacking the columns *x*_1_, …, *x*_*p*_, then an orthonormal basis *N* for the normal to the tangent space has *p* vectors of the form


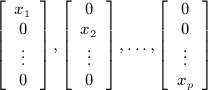


and 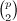 vectors of the form


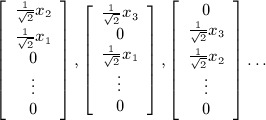


For an arbitrary vector 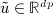, the projection onto the tangent space is then


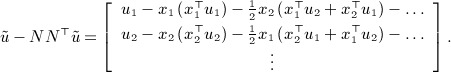


This can be more easily written in matrix form: for an arbitrary 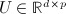, the orthogonal projection onto the Stiefel manifold is


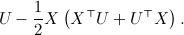


The geodesic flows are more complicated than the spherical case. For *p* > 1, they are no longer simple rotations but can be expressed in terms of matrix exponentials (Edelman *et al.*, 1999, page 310)


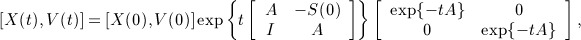


where *A* = *X*(*t*)^⊤^ *V* (*t*) is a skew-symmetric matrix that is constant over the geodesic, and *S*(*t*) = *V* (*t*)^⊤^ *V* (*t*) is non-negative definite.

Although matrix exponentials can be quite computationally expensive, we note that the largest exponential of these is of a 2*p* × 2*p* matrix, which requires *O*(*p*^3^) operations. Other than this and the evaluations of the log-density and its gradients, all the other operations are simple matrix additions and multiplications, the largest of which can be performed in *O*(*dp*^2^) operations; hence, the algorithm scales linearly with *d*.

For the orthogonal group 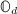, the geodesics have the simpler form (Edelman *et al.*, 1999, equation 2.14)


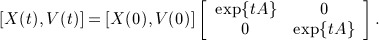


As *A* is skew-symmetric, Rodrigues’ formula gives an explicit form of exp{*tA*} when *d* = 3 in terms of simple trigonometric functions, and this can be extended into higher dimensions (Gallier and Xu, 2002; Cardoso and Leite, 2010).

### 4.4. Product manifolds

Given two manifolds 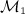 and 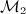, their Cartesian product


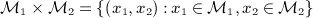


is also a manifold.

Product manifolds arise naturally in many statistical problems; for example, extensions of the von Mises distributions to 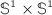 (a torus) have been used to model molecular angles (Singh *et al.*, 2002), and the network eigenmodel in section 5.3 has a posterior distribution on 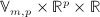.

The geodesics of a product manifold are of the form (*γ*_1_,*γ*_2_), where each *γ*_*i*_ is a geodesic of 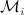. Likewise, the tangent vectors are of the form (*v*_1_,*v*_2_), where each *v*_*i*_ is a tangent to 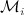. Consequently, for an arbitrary vector (*u*_1_,*u*_2_), the orthogonal projection onto the tangent space is 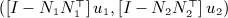, where *N*_*i*_ is an orthonormal basis of 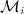.

As a result, when implementing our geodesic Monte Carlo scheme on a product manifold, the key operations (addition of gradient, projection and geodesic update) can be essentially performed in parallel, the only operations requiring knowledge of the other variables being the computation of the log-density and its gradient. Moreover, when tuning the algorithm, it is possible to choose different *ε* values for each constituent manifold, which can be helpful when variables have different scales of variation.

## 5. Illustrative examples

### 5.1. Bingham–von Mises–Fisher distribution

The BVMF distribution is the exponential family on 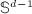 with linear and quadratic terms, with density of the form


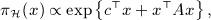


where *c* is a vector of length *d*, and *A* is a *d* × *d* symmetric matrix (Mardia and Jupp, 2000, section 9.3.3).

The Bingham distribution arises as the special case where *c* = 0: this is an axially bimodal distribution, with the modes corresponding to the eigenvector of the largest eigenvalue. The BVMF distribution may or may not be bimodal, depending on the parameter values.

Hoff (2009) develops a Gibbs-style method for sampling from BVMF distribution by first transforming *y* = *E*^⊤^ *x*, where *E*^⊤^ Λ*E* is the eigendecomposition of *A*. Each element *y*_*i*_ of *y* is updated in random order, conditional 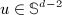, where 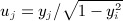 for *j* ≠ *i*. The *y*_*i*_ ∣ *u* are sampled using a rejection sampling scheme with a beta envelope; however, as noted by Brubaker *et al.* (2012), this can give exponentially poor acceptance probabilities (of the order of 10^− 100^) for certain parameter values, particularly when *c* is large in the direction of the negative eigenspectra.

**Fig. 4:**
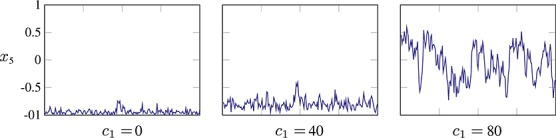
Trace plots of *x*_5_ from 200 samples from the spherical geodesic Monte Carlo sampler (with parameters *ε* = 0.01,*T* = 20) for a Bingham–von Mises–Fisher distribution, with parameters *A* = diag( − 20, − 10,0,10,20) and *c* = (*c*_1_,0,0,0,0). When the distribution is bimodal ( *c*_1_ = 0,40), the sampler has difficulty moving between the modes.

Implementing our geodesic sampling scheme for the BVMF distribution is straightforward, as the gradient of the log-density is simply *c* + 2*Ax*, and extremely fast to run, with run times that are independent of the parameter values. However, as with any gradient-based method, it has difficulty switching between multiple modes (Fig. 4).

A common method of alleviating this problem is to utilize tempering schemes (Neal, 2011, section 5.5.7): these operate by sampling from a class of ‘higher temperature’ distributions with densities of the form


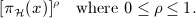


**Fig. 5:**
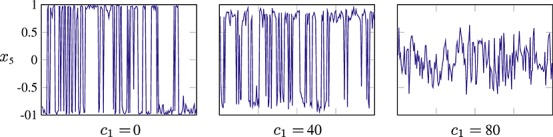
Trace plots of a simulated tempering scheme applied to the target of Fig. 4, using 10 parallel chains to transition between multiple modes. The values of *ρ* were 0.1,0.2, …, 1.0, and 10 random exchanges were applied between parallel geodesic Monte Carlo updates.

Note that this constitutes a simple linear scaling of the log-density and so can be easily incorporated into our method. *Parallel tempering* (Geyer, 1991; Liu, 2008, section 10.4) utilizes multiple chains, each targeting a density with a different temperature. The scheme operates by alternately updating the individual chains, which can be performed in parallel, and randomly switching the values of neighbouring chains with a Metropolis–Hastings correction to maintain detailed balance. The results of utilizing such a scheme are shown in Fig. 5

### 5.2. Non-conjugate simplex models

We can use the transformation to the sphere to sample from distributions on the simplex Δ^*d* − 1^. These arise in many contexts, particularly as prior and posterior distributions for discrete-valued random variables such as the multinomial distribution.

If each observation *x* from the multinomial is completely observed, then the contribution to the likelihood is then *θ*_*x*_, giving a full likelihood of at most *d* terms of form


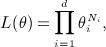


which is conjugate to a Dirichlet prior distribution.

Complications arise if observations are only partially observed. For example, we may have *marginal* observations, which are only observed to a set *S*, in which case the likelihood term is 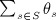, or *conditional* observations, where the sampling was constrained to occur within a set *T*, with likelihood term 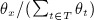. These terms destroy the conjugacy and make computation very difficult.

Such models arise under a *case-cohort design* (Le Polain deWaroux *et al.*, 2012), the risk factors of a particular disease: for the case sample, the risk factors are observed conditional on the person having the disease, and for the cohort sample, the risk factors are observed marginally (as disease status is unknown). Overall population statistics may provide some further information as to the marginal probability of the disease.

The hyperdirichlet R package (Hankin, 2010) provides an interface and examples for dealing with this type of data. We consider the volleyball data from this package: the data arise from a sports league for nine players, where each match consists of two disjoint teams of players, one of which is the winner. The probability of a team *T*_1_ beating *T*_2_ is assumed to be


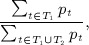


where *p* = (*p*_1_, …, *p*_9_) ∈ Δ^8^. We compare three different methods in sampling from the posterior distribution for *p* under a Dirichlet (*α***1**) prior, for different values of *α*. The results are presented in Table 1.

**Table 1:** Average effective sample size (ESS) across coordinates per 100 samples, and per second, of the Volleyball model under a Dirichlet (*α*1) prior from 1000000 samples. For all samplers, *ε* = 0.01, and for the HMC algorithms, *T* = 20 integration steps were used. We attempted some tuning of the parameters but were unable to obtain any noticeable changes in performance

	*α* = 0.1		*α* = 0.5		*α* = 1.0		*α* = 5.0	
				
	ESS %	ESS/second	ESS %	ESS/second	ESS %	ESS/second	ESS %	ESS/second
RW-MH	0.0064	6.10	0.113	71.1	0.36	158	0.84	290
Spherical RW	0.0089	2.48	0.143	37.6	0.19	51	0.45	123
Simplex HMC	0.0034	0.0079	0.037	0.12	53.4	611	75.6	976
Spherical HMC	0.0187	0.327	77.3	1374	92.6	1616	187.4	3262

The first is a simple random-walk Metropolis–Hastings algorithm. To ensure that the planar constraint 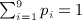 is satisfied, the proposals are made from a degenerate N (*x*,*ε*^2^ [*I* − *nn*^⊤^ ]), where *n* = *d*^− 1 / 2^**1** is the normal to the simplex.

The second is a random walk on the sphere, based on the square root transformation to the sphere from example 2.4, using proposals of the form


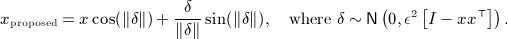


Although this only defines the distribution on the positive orthant, we can extend this distribution to the entire sphere by reflecting about the axes (because we only require knowledge of the density up to proportionality, we can ignore the fact that it is now 2^*d*^ times larger). One benefit of this transformation is that the surface is now smooth and without boundaries, so proposals outside the positive orthant can be accepted.

The third is the geodesic Monte Carlo algorithm on the simplex. We can ensure that the planar constraint is satisfied via the affine constraint methods in 4.1; however, we need to further ensure that the integration paths satisfy the positivity constraints, which can be achieved by reflecting the path whenever it violates the constraint (see the Appendix for further details).

The fourth is our proposed geodesic scheme based on the spherical transformation. As the integrator does not pass any boundaries, no reflections are required.

For small values of *α*, both geodesic Monte Carlo samplers perform poorly, due to the concentration of the density at the boundaries. These peaks cause particular problems for the Hamiltonian-type algorithms, as the discontinuous gradients mean that the integration paths give poor approximations to the true Hamiltonian paths, resulting in poor acceptance probabilities. Moreover, for the algorithm on the simplex, the frequent reflections add to the computational cost.

However, when *α* = 0.5, the spherical geodesic sampler improves markedly: recall from example 2.6 and Fig. 3 that the Dirichlet (0.5) prior is uniform on the sphere, giving continuous gradients. On the simplex, however, the density remains peaked at the boundaries. The simplex sampler improves considerably for values of *α* ≥ 1 (where the gradient is now flat or negative); however the spherical algorithm still retains a slight edge. Interestingly, the spherical random walk sampler performs poorly in all of the examples.

### 5.3. Eigenmodel for network data

We use the network eigenmodel of Hoff (2009) to demonstrate how Stiefel manifold models can be used for dimension reduction and how our geodesic sampling scheme may be used for Stiefel and product manifolds. This is a model for a graph on a set of *m* nodes, where for each unordered pair of nodes {*i*,*j*}, there is a binary observation *Y*_{*i*,*j*}_ indicating the existence of an edge between *i* and *j*.

The specific example of Hoff (2009) is a protein interaction network, where for *m* = 270 proteins, the existence of the edge indicates whether or not the pair of proteins interact.

The model represents the network by assuming a low (*p* = 3) dimensional representation for the probability of an edge


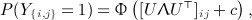


where 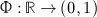 is the probit link function, *U* is an orthonormal *m* × *p* matrix and Λ is a *p* × *p* diagonal matrix. *U* is assumed to have a uniform prior distribution on 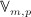 (with respect to the Hausdorff measure), the diagonal elements of Λ have a N (0,*m*) distribution and *c* ∼ N (0,10^2^).

Hoff (2009) uses column-wise Gibbs updates for sampling *U*, exploiting the fact that the probit link provides an augmentation that allows these to be sampled as a BVMF distribution. However, as mentioned in section 4.3, this requires computing the full null space of *U* at each iteration.

**Fig. 6:**
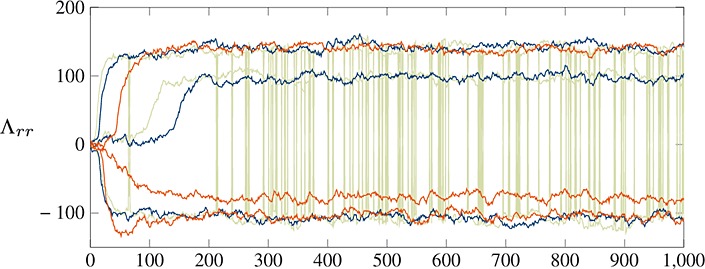
Trace plots of 1000 samples of the diagonal elements of Λ from geodesic Monte Carlo sampler on the network eigenmodel. One chain (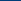) converges to the same mode as Hoff (2009), while the other (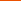) converges to a local mode, with approximately 10^− 36^ of the density. By incorporating this into a parallel tempering scheme (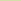), the sampler rapidly finds the higher mode and is able to switch between the various permutations.

We implement geodesic Monte Carlo on the product manifold, details of which are given in the Appendix. Trace plots from two chains of the diagonal elements of Λ appear in Fig. 6: note that one chain appears to get stuck in a local mode, while the other converges to the same as the method of Hoff (2009).

By incorporating this approach into a parallel tempering scheme, the model is able to find the larger mode with greater reliability. Moreover, unlike the algorithm of Hoff (2009), it is capable of switching between the permutations of Λ, which would further suggest that this is indeed the global mode.

## 6. Conclusion and discussion

We have presented a scheme for sampling from distributions defined on manifolds embedded in Euclidean space by exploiting their known geodesic structure. This method has been illustrated using applications from directional statistics, discrete data analysis and network analysis. This method does not require any conjugacy, allowing greater flexibility in the choice of models: for instance, it would be straightforward to change the probit link in section 5.3 to a logit. Moreover, when used in conjunction with a tempering scheme, it is capable of efficiently exploring complicated multimodal distributions.

Our approach could be widely applicable to problems in directional statistics, such as the estimation of normalization constants that are often otherwise numerically intractable. The method of transforming the simplex to the sphere could be useful for applications dealing with high-dimensional discrete data, such as statistical genetics and language modelling. Stiefel manifolds arise naturally in dimension reduction problems, and our methods could be particularly useful where the data are not normally distributed, for instance the analysis of survey data with discrete responses, such as Likert scale data. Furthermore, this method could be utilized in statistical shape and image analysis for determining the orientation of objects in projected images.

The major constraint of this technique is the requirement of an explicit form for the geodesic flows that can be easily evaluated numerically. These are not often available; for instance, the geodesic paths of ellipsoids require often computationally intensive elliptic integrals.

Of the examples we consider, the geodesics of the Stiefel manifold case are the most demanding, due to the matrix exponential terms. An alternative approach would be to utilize a Metropolis-within-Gibbs style scheme over subsets of columns, for example by updating a pair of columns such that they remain orthogonal to the remaining columns.

However, once the geodesics and the orthogonal tangent projection of the manifold are known, the remaining process of computing the derivatives is straightforward, and could be easily implemented using automatic differentiation tools, as is used in the Stan MCMC library (Stan Development Team, 2012), currently under development.

## Appendix A: Reflecting at boundaries of the simplex

Neal (2011, section 5.5.1.5) notes that when an integration path crosses a boundary of the sample space, it can be reflected about the normal to the boundary to keep it within the desired space. He considers boundaries that are orthogonal to the *i*th axis, with normals of the form *δ*_*i*_. Whenever such a constraint is violated, the position and velocity are replaced by

where *b*_*i*_ is the boundary (either upper or lower). As no other coordinates are involved in this reflection, this can be performed in parallel for all constrained coordinates.

However, for the simplex Δ^*d* − 1^, the normals are not of the form *δ*_*i*_, as this would result in the path being reflected off the plane . Instead, we need to reflect about the projection of *δ*_*i*_ onto the plane, that is,

A procedure for performing the position updates is given in Algorithm 2.

**Table:**

Unfortunately, this procedure cannot be applied to the RMHMC integrator proposed by Girolami & Calderhead (2011), as the implicit steps involved make it difficult to calculate the reflections.

## Appendix B: Network eigenmodel

Define the *p* × *p* symmetric matrices *η* = *U*Λ*U*^⊤^ + *c* and *Y* ^*^, where

Then using the property that 1 − Φ(*x*) = Φ( − *x*), the log-density of the posterior is

The gradients with respect to the parameters are

where the gradients with respect to the linear predictors are

The programme was implemented in MATLAB (The MathWorks Inc., Natick, Massachusetts, USA). To avoid numerical overflow errors, the ratio *φ*(*x*) / Φ(*x*), as well as logΦ(*x*) for negative values of *x*, are calculated using the erfcx function. The matrix exponential terms were calculated using the inbuilt expm function, which utilizes a Padé approximation with scaling and squaring.

Different *ε* values were used for each parameter: *ε*_*U*_ = 0.005, *ε*_Λ_ = 0.1 and *ε*_*c*_ = 0.001. *T* = 20 integration steps were run for each iteration. The parallel tempered version utilized 20 parallel chains, with 10 proposed exchanges between parallel updates.
